# Evaluate the effects of statins on myocardial remodeling in ApoE-/- mice at 3T cardiac magnetic resonance

**DOI:** 10.1186/1532-429X-17-S1-P83

**Published:** 2015-02-03

**Authors:** Huan Zhang, Xiaohai Ma, Zuoguang Wang, Lei Zhao, Chen Zhang, Zhanming Fan

**Affiliations:** 1 Beijing Anzhen Hospital, Capital Medical University, Beijing, China

## Background

Noninvasively evaluate the cardiac remodeling and the effect of statins on cardiac remodeling are important for understanding this physiopathologic process. Therefore, we try to use the cardiac magnetic resonance (CMR) to observe the progression of cardiac morphology and function in Apolipoprotein E-deficient (ApoE-/-) mice fed with normal and high-fat diet separately, and evaluate the effect of statins on regression of cardiac remodeling.

## Methods

Thirty six male ApoE-/- mice at the age of eight weeks were randomly divided into two groups, fed with normal and high-fat diet for 16 weeks respectively. Then six mice of each group were randomly executed for pathologic examination. Rest mice in each group were randomized divided into two sub-groups, model group and statins intervention group with 6 mice in each group. Normal C57BL/6J mice were enrolled as the control group. The CMR was performed with morphological examination at the 8^th^, 16^th^ and 40^th^ weeks. Left ventricular (LV) wall thickness and function indices (end-systolic volume, end-diastolic volume, ejection fraction, left ventricular mass) were measured. Serum cholesterol, myocardial cholesterol, nitric oxide (NO), superoxide dismutase (SOD), malondialdehyde (MDA) was sampled and analyzed after each time of CMR examination.

## Results

The LV wall thickness which determined with CMR was correlated well with pathological results (P<0.05). Along with the weeks increased, the LV wall thickness, serum cholesterol and MDA were significantly increased (P<0.05), cardiac function, NO and SOD were gradually decreased (P<0.05) in model group, while in statins intervention group, the LV wall thickness, serum cholesterol and MDA were decreased (P<0.05), cardiac function, NO and SOD were increased (P<0.05).

## Conclusions

The progression and regression of cardiac morphology and function of ApoE-/- mice could be evaluated at 3T CMR qualitatively and quantitatively. CMR can be used for monitoring the dynamic changes of cardiac remodeling noninvasively and accurately.

## Funding

The national natural science foundation of China.

